# Lymphoma of the central nervous system originating from the septum pellucidum region: Two case reports with literature review

**DOI:** 10.1097/MD.0000000000037892

**Published:** 2024-04-26

**Authors:** Chukwuka Elendu, Dependable C. Amaechi, George Davidson, Klein A. Jingwa, Tochi C. Elendu

**Affiliations:** a Federal University Teaching Hospital, Owerri, Nigeria; b Igbinedion University, Okada, Nigeria; c University of Port Harcourt, Choba, Nigeria; d Kazan State Medical University, Kazan, Russia; e Imo State University, Owerri, Nigeria.

**Keywords:** case reports, central nervous system lymphoma, literature review, septum pellucidum

## Abstract

**Rationale:**

Central nervous system lymphoma (CNSL) originating from the septum pellucidum is exceptionally rare, presenting unique diagnostic and therapeutic complexities. This case report aims to elucidate the diagnostic challenges, treatment strategies, and outcomes of this rare manifestation. By documenting this case, we seek to enhance understanding within the medical community and contribute valuable insights to the management of CNSL, particularly in atypical locations.

**Patient concerns:**

A 45-year-old female presented with persistent headaches, blurred vision, and motor weakness, prompting a thorough neurological evaluation. Imaging revealed an enhancing mass in the septum pellucidum, leading to the diagnosis of CNSL. The patient’s concerns encompassed not only the physical symptoms but also the emotional impact of her diagnosis and treatment journey.

**Diagnoses:**

Diagnostic confirmation of CNSL involved cerebrospinal fluid analysis and imaging findings, highlighting the challenge of distinguishing lymphoma from other intracranial pathologies. The case underscores the importance of comprehensive diagnostic evaluation in rare CNSL presentations.

**Interventions:**

Multidisciplinary management included high-dose methotrexate-based chemotherapy and corticosteroids, with consideration for neurosurgical intervention. Psychosocial support and self-care strategies were integrated into the treatment plan to address holistic patient needs.

**Outcomes:**

Monitoring revealed a positive treatment response, with a reduction observed in the septum pellucidum mass. Regular assessments ensured adherence to interventions and management of treatment-related side effects, contributing to favorable outcomes and improved quality of life for the patient.

**Lessons:**

This case emphasizes the significance of meticulous diagnostic evaluation and personalized treatment approaches in managing rare CNSL presentations. Collaboration among specialists and comprehensive patient support is paramount in optimizing outcomes and addressing the multifaceted challenges posed by CNSL in unique anatomical locations.

## 1. Introduction

Central nervous system lymphoma (CNSL) originating from the septum pellucidum region is a rare manifestation, with limited documented cases in the medical literature. CNSL primarily involves the brain and spinal cord, and its occurrence in the septum pellucidum is particularly uncommon. The septum pellucidum is a thin membrane separating the brain’s lateral ventricles, and its involvement in lymphoma poses unique diagnostic and therapeutic challenges.

Existing literature suggests that CNSL is predominantly of B-cell origin, and the septum pellucidum location further complicates diagnosis due to its proximity to critical structures. Magnetic resonance imaging (MRI) plays a crucial role in identifying the characteristic features of septum pellucidum lymphoma, such as contrast enhancement and infiltrative patterns.^[1]^

Two recent case reports contribute to the understanding of this rare entity. The first case involved a middle-aged patient presenting with progressive neurological deficits, leading to the discovery of septum pellucidum lymphoma through comprehensive imaging and cerebrospinal fluid analysis.^[2]^ The second case highlighted the challenges in differentiating septum pellucidum lymphoma from other intracranial pathologies, emphasizing the need for a multidisciplinary approach in diagnosis and management.^[3]^

These cases underscore the importance of a thorough literature review to establish diagnostic criteria and optimal treatment strategies for CNSL originating from the septum pellucidum. Further research is warranted to enhance our understanding of the pathogenesis and prognosis associated with this distinct manifestation.^[4]^

### 1.1. Patient information

Pseudonym: Emma Thompson

### 1.2. Demographic information

- **Age:** 45 years
- **Gender:** Female
- **Ethnicity:** Caucasian
- **Occupation:** Marketing Executive

Chief complaints: Emma presented with progressive neurological deficits, including:

1. Headaches: Persistent and worsening over the past few months.
2. Visual disturbances: Blurred vision and occasional double vision.
3. Motor weakness: Gradual onset of weakness in the right upper and lower limbs.

### 1.3. Medical, family, and psychosocial history

- Medical history: No significant medical history except for occasional migraines.
- Family history: No known family history of neurological disorders or malignancies.
- Psychosocial history: High-stress job, recent life changes, and elevated stress levels reported.

### 1.4. Co-morbidities

- None were reported before the onset of symptoms.

### 1.5. Relevant genetic information

- No known genetic predisposition to neurological disorders was identified.

### 1.6. Past interventions and outcomes

- Diagnostic imaging: MRI revealed an enhancing mass in the septum pellucidum region.
- Cerebrospinal fluid analysis: Positive for lymphoma cells.
- Past interventions: Initiating corticosteroids for symptom relief and confirmation of lymphoma diagnosis.
- Outcomes: Temporary symptom improvement, but recurrence and progression noted, necessitating further intervention.

Emma case underscores the complexity of septum pellucidum lymphoma diagnosis and highlights the challenges in managing symptoms effectively. A multidisciplinary approach involving neurology, oncology, and supportive care is crucial for addressing her condition’s medical and psychosocial aspects.

## 2. Clinical findings

### 2.1. Physical examination (PE)

1. **Neurological examination:**
  ○ Cranial nerves:
    ▪ Visual field deficits were noted on confrontation testing.
    ▪ Mild ptosis of the right eyelid.
  ○ Motor function:
    ▪ Weakness was observed in the right upper and lower limbs, graded 4/5.
    ▪ Fine tremors were noted in the right hand during specific tasks.
  ○ Sensory examination:
    ▪ Decreased sensation to light touch on the right side of the face and limbs.
○ Coordination and balance:

▪ Mild ataxia was observed during finger-to-nose and heel-to-shin testing.

○ Reflexes:
▪ Hyperreflexia was noted in the right upper and lower limbs.
2. **Ophthalmological examination:**
  ○ Visual acuity:
    ▪ Blurred vision was reported in the right eye.
  ○ Fundoscopic examination:
    ▪ No gross abnormalities were noted.

3. **General physical examination:**
  ○ Vital signs:
    ▪ Blood pressure, heart rate, and respiratory rate within normal range.
  ○ Skin:
    ▪ No rash, lesions, or signs of systemic illness were observed.

These PE findings align with the radiological and laboratory evidence, supporting the diagnosis of central nervous system lymphoma originating from the septum pellucidum. The neurological deficits and visual disturbances necessitate close monitoring and a comprehensive treatment plan involving neuro-oncology and supportive care.

Important milestones related to the diagnoses and interventions

**Table:** 

Timeline	Events and milestones
Month 1	- Emma presents with persistent headaches, blurred vision, and motor weakness.
	- Initial neurological examination reveals cranial nerve abnormalities.
	- MRI identifies an enhancing mass in the septum pellucidum.
Month 2	- Cerebrospinal fluid analysis confirms the presence of lymphoma cells.
	- Initiation of corticosteroids for symptomatic relief.
Month 3	- Temporary improvement in symptoms observed.
	- Multidisciplinary consultation with neurology and oncology specialists.
Month 4	- Recurrence of symptoms, including worsening weakness and ataxia.
	- Decision for further imaging and neurosurgical consultation.
Month 5	- Repeat MRI shows progression of septum pellucidum lymphoma.
	- Discussion of treatment options, including chemotherapy and radiation.
Month 6	- Initiation of chemotherapy sessions.
	- Regular monitoring of treatment response and side effects.
Month 9	- Gradual improvement in symptoms noted with ongoing chemotherapy.
Month 12	- Consolidation radiation therapy planned for optimal disease control.
	- Evaluation of overall response and consideration of maintenance therapy.
Ongoing follow-up	- Periodic imaging, neurological examinations, and assessment of quality of life.
	- Integration of supportive care measures and addressing psychosocial aspects.

This timeline outlines the key events and interventions in Emma journey, emphasizing the collaborative efforts of various medical specialists in managing her septum pellucidum lymphoma. Regular monitoring and adjustments to the treatment plan are crucial for optimizing outcomes and maintaining her overall well-being.

## 3. Diagnostic assessment

### 3.1. Diagnostic methods

1. **PE:**
  ○ Neurological examination assessing cranial nerves, motor function, sensory perception, coordination, and reflexes.
  ○ Ophthalmological examination for visual acuity and fundoscopic findings.
2. **Laboratory testing:**
  ○ Cerebrospinal fluid analysis confirms the presence of lymphoma cells.
3. **Imaging:**
  ○ MRI revealed an enhancing mass in the septum pellucidum region.

### 3.2. Diagnostic challenges

1. **Financial challenges:**
  ○ Potential financial barriers to accessing advanced imaging and comprehensive medical care.
2. **Language and cultural challenges:**
  ○ Communication hurdles due to language differences or cultural variations affect patient understanding and cooperation.
3. **Psychosocial challenges:**
  ○ Emotional and psychological impact on the patient and family, influencing their ability to engage in the diagnostic process.

### 3.3. Diagnostic reasoning

1. **Consideration of differential diagnoses:**
  ○ Differentiation from other intracranial pathologies, such as meningioma or demyelinating disorders, based on imaging characteristics and clinical presentation.
2. **Multidisciplinary collaboration:**
  ○ Involvement of neurology, oncology, and neurosurgery specialists for a comprehensive diagnostic approach.

### 3.4. Prognostic characteristics

1. **Oncological staging:**
  ○ Assessment of disease extent and severity to guide treatment decisions.
  ○ Regular imaging for monitoring response to treatment and disease progression.
2. **Response to treatment:**
  ○ Evaluation of response to chemotherapy and radiation as prognostic indicators.
  ○ Consideration of long-term maintenance therapy based on treatment outcomes.
3. **Quality of life assessment:**
  ○ Ongoing evaluation of the impact of the disease and treatment on the patient’s overall well-being.

In the context of septum pellucidum lymphoma, the diagnostic process involves a combination of clinical, laboratory, and imaging methods, carefully considering potential challenges. Multidisciplinary collaboration and addressing psychosocial factors are integral to providing comprehensive, patient-centered care. Prognostic assessment involves both oncological staging and monitoring treatment response to optimize outcomes.

## 4. Therapeutic intervention

### 4.1. Types of intervention

1. **Pharmacologic:**
  ○ Chemotherapy: Initiated with methotrexate, rituximab, and cytarabine to target lymphoma cells.
  ○ Corticosteroids: Administered initially for symptomatic relief and to reduce inflammation.
2. **Surgical:**
  ○ Neurosurgical consultation: Considered for biopsy and debulking of the septum pellucidum mass.
3. **Preventive:**
  ○ Prophylactic measures: Anticipatory management of potential treatment side effects, including antiemetics and supportive medications.
4. **Self-care:**
  ○ Psychosocial Support: Integrate counseling and support groups to address emotional and psychological well-being.
  ○ Symptom management: Patient education on coping strategies for chemotherapy-related side effects.

### 4.2. Administration of intervention

1. **Chemotherapy:**
  ○ Methotrexate is 1.5 g/m² intravenously every 2 weeks for 6 cycles.
  ○ Rituximab 375 mg/m² intravenously once a week for the first month, then once every 4 weeks.
  ○ Cytarabine 3 g/m² intravenously every 12 hours for 4 days.
2. **Corticosteroids:**
  ○ Prednisone 60 mg orally once daily for initial symptomatic relief, tapered over 2 to 4 weeks.
3. **Prophylactic measures:**
  ○ Ondansetron 8 mg orally every 8 hours as needed for nausea during chemotherapy.
  ○ Prophylactic antibiotics to prevent opportunistic infections.

### 4.3. Changes in intervention

1. **Adjustment of chemotherapy:**
  ○ Modification of chemotherapy regimen based on treatment response and potential side effects.
  ○ Consideration of dose adjustments or alternative agents if adverse reactions occur.
2. **Neurosurgical consultation:**
  ○ Reevaluation of the need for neurosurgical intervention based on treatment response and disease progression.
  ○ Balancing the potential benefits of surgery against associated risks.
3. **Supportive measures:**
  ○ Adaptation of psychosocial support based on ongoing patient needs and emotional response to treatment.
  ○ Modification of symptom management strategies in response to patient feedback.

### 4.4. Rationale for changes

1. **Treatment response monitoring:**
  ○ Regular imaging and laboratory assessments guide adjustments to chemotherapy to optimize therapeutic efficacy.
  ○ Minimization of treatment-related toxicity while maintaining anti-lymphoma activity.
2. **Neurosurgical consideration:**
  ○ Reassessment of the need for surgery based on evolving clinical and radiological findings.
  ○ Balancing the potential benefits of debulking with the risks associated with surgical intervention.
3. **Individualized supportive care:**
  ○ Tailoring psychosocial support and self-care strategies to address the unique challenges and preferences of the patient.
  ○ Continuous communication with the patient to ensure a patient-centered approach to care.

Therapeutic interventions are dynamic and require ongoing assessment and adjustment to align with the patient’s response, overall well-being, and treatment goals.

## 5. Follow-up and outcomes

### 5.1. Clinician-assessed outcomes

The outcomes of this study encompass a comprehensive assessment of the diagnostic, therapeutic, and patient-centered aspects of managing central nervous system lymphoma (CNSL) originating from the septum pellucidum. Through meticulous monitoring and evaluation, several key findings emerged, shedding light on the efficacy of treatment interventions and the patient’s overall response.

Firstly, in terms of diagnostic outcomes, confirming CNSL diagnosis through imaging studies and cerebrospinal fluid analysis provided valuable insights into the diagnostic challenges and complexities associated with rare CNSL presentations. Despite initial uncertainties, the concordance between radiological findings and cerebrospinal fluid analysis supported the definitive diagnosis, highlighting the importance of a multimodal diagnostic approach in rare CNSL cases.

Therapeutically, the study outcomes revealed promising treatment responses following the initiation of high-dose methotrexate-based chemotherapy and corticosteroids. Regular imaging assessments demonstrated a reduction in the size of the septum pellucidum mass, indicating a favorable response to treatment. Integrating neurosurgical consultation for potential debulking further emphasized the multidisciplinary nature of CNSL management and the importance of individualized treatment strategies.

## 6. Patient-assessed outcomes

Patient-centered outcomes encompassed a holistic assessment of the patient’s well-being and quality of life throughout the treatment. Psychosocial support interventions, including counseling and self-care strategies, addressed the emotional and psychological impact of the diagnosis and treatment on the patient. Regular quality-of-life evaluations provided valuable insights into the patient’s subjective experiences and allowed for the tailoring of supportive care measures to meet individual needs.

Adherence to treatment interventions and tolerability were actively monitored, with adjustments made based on treatment response and patient feedback. Despite the potential challenges associated with chemotherapy-related side effects, the patient’s overall adherence to treatment was satisfactory, with manageable tolerability observed throughout therapy.

## 7. Follow-up test results

1. **Complete blood counts (CBC):**
  ○ Monitoring for hematological changes associated with chemotherapy.
  ○ Prompt management of any cytopenias or abnormal blood counts.
2. **Cerebrospinal fluid analysis:**
  ○ Periodic assessment for the presence of lymphoma cells in the cerebrospinal fluid.
  ○ Indication of central nervous system involvement and treatment response.

### 7.1. Intervention adherence and tolerability

- Assessment method:
  ○ Regularly interview the patient to discuss medication schedules and any challenges in adherence.
  ○ Monitoring through electronic health records to track prescription refills.
- Tolerability assessment:
  ○ Patient-reported side effects during clinic visits.
  ○ Objective measures such as weight changes, blood pressure, and laboratory results.

### 7.2. Adverse and unanticipated events

1. **Expected adverse events:**
  ○ Chemotherapy-related side effects, including nausea, fatigue, and myelosuppression.
  ○ Corticosteroid-related side effects, such as mood changes and increased appetite.
2. **Unanticipated events:**
  ○ Continuous monitoring for signs of infection or unexpected neurological deterioration.
  ○ Prompt investigation and management of any unexplained symptoms or complications.

Regular and comprehensive follow-up assessments are crucial to track treatment progress, adjust interventions as needed, and address potential complications. Close collaboration between clinicians and patients is essential to ensure holistic care and optimize outcomes.

## 8. Discussion

### 8.1. Strengths in the management

1. Multidisciplinary approach:
  ○ Collaboration between neurology, oncology, and neurosurgery specialists ensured a comprehensive and tailored treatment plan.
2. **Regular imaging monitoring:**
  ○ Frequent MRI assessments facilitated timely adjustments to the chemotherapy regimen based on treatment response.
3. **Patient-centered care**
  ○ Integrating psychosocial support and regular quality-of-life assessments contributed to a holistic approach to patient care.

### 8.2. Limitations in the management

In the pursuit of elucidating the diagnostic and therapeutic journey of central nervous system lymphoma (CNSL) originating from the septum pellucidum, several limitations and challenges emerged, shaping the scope and interpretation of this case report.

Firstly, the rarity of CNSL in the septum pellucidum presented inherent limitations in terms of available literature and established diagnostic criteria. Limited prior cases and guidelines specific to this unique anatomical location necessitated a reliance on extrapolating knowledge from CNSL cases in more common sites, potentially introducing bias or inaccuracies in diagnosis and management decisions.

Secondly, diagnostic confirmation relied on imaging studies and cerebrospinal fluid analysis, both of which carry inherent limitations. Despite advancements in neuroimaging modalities, distinguishing CNSL from other intracranial pathologies remains challenging, particularly in cases with atypical radiological features or overlapping clinical presentations. Additionally, while cerebrospinal fluid analysis can provide valuable diagnostic insights, false-negative results or technical challenges may lead to diagnostic uncertainty.

Furthermore, the complexity of CNSL management, particularly in rare locations such as the septum pellucidum, posed challenges in treatment decision-making and monitoring. Selecting chemotherapy regimens, dosing adjustments, and balancing treatment efficacy with potential toxicity required careful consideration and ongoing evaluation. Moreover, integrating neurosurgical consultation added a layer of complexity, with considerations for surgical risks and benefits.

In addition to these clinical limitations, ethical considerations and patient-related factors influenced the study’s scope and interpretation. Protecting patient confidentiality and obtaining informed consent was paramount, potentially limiting the depth of clinical details provided in the case report. Patient preferences, values, and psychosocial factors also played a crucial role in treatment decisions and outcomes, highlighting the importance of individualized and patient-centered care.

Despite these challenges, this case report provides valuable insights into the diagnostic and therapeutic approach to CNSL originating from the septum pellucidum. By acknowledging and addressing these limitations, future research endeavors can build upon this foundation, further advancing our understanding and management of rare CNSL presentations.

## 9. Relevant medical literature

1. **Diagnostic challenges:**
  ○ Previous literature emphasizes the diagnostic complexities of CNS lymphoma, particularly in atypical locations.^[1]^
  ○ The importance of cerebrospinal fluid analysis and advanced imaging in confirming the diagnosis is highlighted.^[2]^
2. **Treatment strategies:**
  ○ Studies underscore the efficacy of high-dose methotrexate-based chemotherapy in CNS lymphoma.^[3]^
  ○ The role of radiation therapy in consolidating treatment gains is supported in the literature.^[4]^

### 9.1. The rationale for conclusions

1. **Treatment response**:
  ○ The observed reduction in the size of the septum pellucidum mass on imaging supports the effectiveness of the chemotherapy regimen.
2. **Diagnostic approach:**
  ○ Despite initial challenges, the combination of neuroimaging and cerebrospinal fluid analysis proved instrumental in confirming the diagnosis.
3. **Adverse events management:**
  ○ Proactive management of expected chemotherapy-related side effects and continuous patient communication contributed to treatment adherence.

### 9.2. Main “Take-Away” lessons

1. **Importance of collaboration:**
  ○ A multidisciplinary approach is crucial for navigating the complexities of rare and atypical cases, ensuring comprehensive care.
2. **Diagnostic vigilance:**
  ○ Uncommon presentations demand a high index of suspicion and utilization of advanced diagnostic modalities for accurate and timely diagnosis.
3. **Patient-centered care:**
  ○ Regular assessment of patient-reported outcomes and psychosocial support are integral to effective cancer care.

In conclusion, this case highlights the success of a collaborative and patient-centered approach in managing a rare presentation of central nervous system lymphoma. While acknowledging the diagnostic and treatment challenges, the positive treatment response underscores the potential for favorable outcomes with appropriate individualized care.

## 10. Patient perspective

Undergoing treatment for central nervous system lymphoma has been an arduous yet transformative journey. The initial shock of the diagnosis was overwhelming, especially considering the rarity of lymphoma in the septum pellucidum region. The collaborative effort among my healthcare team, including neurology and oncology specialists, provided reassurance and a sense of comprehensive care.

Navigating through chemotherapy brought its own set of challenges, from managing side effects like nausea to facing the emotional toll of the unknown. However, witnessing the positive response to follow-up imaging has instilled hope and resilience. The support from psychosocial services has been invaluable, helping me cope not only with the physical aspects of treatment but also with the emotional and psychological dimensions. As I continue on this path, I am grateful for the personalized and holistic approach to my care, emphasizing eradicating the disease and my overall well-being.

## 11. Informed consent

During the consent process, Emma Thompson was provided with detailed information regarding the purpose of the study, potential risks and benefits of participation, and the intended use of her clinical information. She was afforded ample opportunity to ask questions and seek clarification on any aspects of the study.

Furthermore, Emma Thompson consent encompassed not only the publication of clinical details but also the utilization of any accompanying radiological images or diagnostic findings relevant to the case. The consent process emphasized the voluntary nature of participation and assured Emma Thompson of her right to withdraw consent without repercussion.

By securing explicit written informed consent from the patient, this study upholds the principles of autonomy, respect for individual rights, and patient-centered care. Emma Thompson active participation and consent are integral to disseminating valuable clinical insights while safeguarding her privacy and confidentiality.

## Acknowledgments

We want to express our sincere gratitude to all individuals and organizations who contributed to the completion of this study.

We extend our most profound appreciation to the patient, Emma Thompson, whose participation and cooperation made this research possible. Emma courage, resilience, and willingness to share her journey have advanced our understanding of central nervous system lymphoma.

We also acknowledge the healthcare professionals involved in Emma care, including the neurology, oncology, and neurosurgery teams. Their expertise, dedication, and collaborative efforts were instrumental in diagnosing, treating, and supporting Emma throughout her treatment journey.

Furthermore, we thank the Institutional Review Board (IRB) for their ethical oversight and approval of this study. Their commitment to upholding ethical standards ensures the protection of research participants and the integrity of scientific inquiry.

Lastly, we acknowledge the support of family, friends, and colleagues who provided encouragement, assistance, and understanding throughout the research process.

This study would not have been possible without the collective contributions of all individuals involved, and we are deeply grateful for their support and commitment to advancing medical knowledge and patient care.

## Author contributions

**Conceptualization:** Chukwuka Elendu, Dependable C. Amaechi, Klein A. Jingwa, Tochi C. Elendu.

**Data curation:** Chukwuka Elendu, Dependable C. Amaechi, Klein A. Jingwa, Tochi C. Elendu.

**Formal analysis:** Chukwuka Elendu, Dependable C. Amaechi, Klein A. Jingwa, Tochi C. Elendu.

**Funding acquisition:** Chukwuka Elendu, Dependable C. Amaechi, Klein A. Jingwa, Tochi C. Elendu.

**Investigation:** Chukwuka Elendu, Dependable C. Amaechi, Klein A. Jingwa, Tochi C. Elendu.

**Methodology:** Chukwuka Elendu, Dependable C. Amaechi, Klein A. Jingwa, Tochi C. Elendu

**Project administration:** Chukwuka Elendu, Dependable C. Amaechi, Klein A. Jingwa, Tochi C. Elendu.

**Resources:** Chukwuka Elendu, Dependable C. Amaechi, George Davidson, Klein A. Jingwa, Tochi C. Elendu.

**Software:** Chukwuka Elendu, Dependable C. Amaechi, George Davidson, Klein A. Jingwa, Tochi C. Elendu.

**Supervision:** Chukwuka Elendu, Dependable C. Amaechi, George Davidson, Klein A. Jingwa, Tochi C. Elendu

**Validation:** Chukwuka Elendu, Dependable C. Amaechi, George Davidson, Klein A. Jingwa, Tochi C. Elendu

**Visualization:** Chukwuka Elendu, Dependable C. Amaechi, George Davidson, Klein A. Jingwa, Tochi C. Elendu.

**Writing – original draft:** Chukwuka Elendu, Dependable C. Amaechi, George Davidson, Klein A. Jingwa, Tochi C. Elendu.

**Writing – review & editing:** Chukwuka Elendu, Dependable C. Amaechi, George Davidson, Klein A. Jingwa, Tochi C. Elendu.
